# Stoichiometric homeostasis of *Morus alba* in the dry-hot valley

**DOI:** 10.3389/fpls.2025.1520936

**Published:** 2025-03-17

**Authors:** Haixia Guo, Sheng Luo, Siyuan Chen, Yike Li, Jianhua Zhang, Guantao Chen, Xie Wang

**Affiliations:** ^1^ Key Laboratory of the Philosophy and Social Sciences of Sichuan Province on the Monitoring and Evaluation of the Utilization of Rural Land, Chengdu Normal University, Chengdu, China; ^2^ Ecological Restoration and Biodiversity Conservation Key Laboratory of Sichuan Province, Chengdu Institute of Biology, Chinese Academy of Sciences, Chengdu, China; ^3^ Institute of Agricultural Resources and Environment, Sichuan Academy of Agricultural Sciences, Chengdu, China; ^4^ Southwest Key Laboratory of Mountain Agricultural Environment, Ministry of Agriculture and Rural Areas, Chengdu, China

**Keywords:** *Morus alba*, soil, slope, altitude, stoichiometric homeostasis

## Abstract

**Introduction:**

Stoichiometric homeostasis is an important strategy used by plants to function optimally in changing environments.

**Methods:**

In order to investigate whether plants under stricter resource restrictions exhibit stronger homeostasis, this study took *M. alba* inhabiting in a dry-hot valley as the research subject.

**Results:**

The stoichiometry of *M. alba* leaves, their variations in response to altitude and slope, and their correlations with soil were analyzed. The results showed that soil nutrient levels were higher on the shady slope compared to the sunny slope, and responded differently to altitude on the two slopes. On the sunny slope, soil carbon (C) content increased significantly with altitude, whereas on the shady slope, soil phosphorus (P) content decreased with increasing altitude. The C: N and C: P ratios of the soil were lower than the average in China. The C: N and C: P ratios of *M. alba* leaves were lower than those of global and Chinese forest ecosystems. The N: P ratio of *M. alba* leaves was < 14. However, no significant correlation was observed between *M. alba* leaves and soil C, N, P, or stoichiometric characteristics. The changes in C, N, and P and their ratios in *M. alba* leaves did not correspond with those in the soil. *M. alba* exhibited "strict homeostasis" on both sunny and shady slopes.

**Discussion:**

The results suggest that *M. alba's* growth is limited by nutrients availability, particularly nitrogen. The strict stoichiometric homeostasis is an adaptation strategy for *M. alba* in dry-hot valleys to alleviate nutrient limitations, which leads to a decoupling of ecological stoichiometry between *M. alba* leaves and soil.

## 1 Introduction

Ecological stoichiometry examines the balance of multiple chemical elements in ecological interactions, with a focus on C, N, and P (Elser et al., 2000, 2010; Fan et al., 2015). It serves as a powerful tool for understanding plant adaptation strategies to changing environments and detecting nutrient limitations (Güsewell, 2004; Zhou et al., 2024). To function optimally, organisms must maintain a relatively fixed C:N:P ratio, a concept known as stoichiometric homeostasis (Sterner and Elser, 2002; Güsewell, 2004). This homeostatic balance exists at both the individual and community levels (Bertrand et al., 2019). The concept of stoichiometric homeostasis was first introduced by Redfield in 1958, who discovered that the C:N:P ratio of plankton remained consistently at 106:16:1 (Redfield, 1958). Since then, stoichiometric homeostasis has been observed in various organisms, including microbes, forests, and herbaceous plants (Makino et al., 2003; Zhang et al., 2018). However, plant stoichiometry often changes in response to environmental fluctuations, as plants primarily obtain their nutrients from the soil (Reich and Oleksyn, 2004). Wirtz and Kerimoglu (2016) termed this variation as stoichiometric flexibility, identifying it is a strategy autotrophs use to optimize resource utilization under nutrient-limited conditions Stoichiometric flexibility is influenced by various factors, including an organism’s nature, climate, altitude, nutrient availability, intensity of perturbation, and geographic range size (Elser et al., 2010; Sistla and Schimel, 2012; Bertrand et al., 2019). To quantify an organism’s ability to maintain stoichiometric homeostasis or its range of stoichiometric flexibility, Sterner and Elser (2002) proposed a continuously variable regulation parameter (*H*), which was found to vary significantly among different organisms (Elser et al., 2010). Numerous studies have explored stoichiometric flexibility across various organisms and levels, leading to some generalizable hypotheses. For instance, stoichiometric homeostasis tends to be stricter at higher trophic levels compared than at lower ones (Hessen et al., 2004; Sistla and Schimel, 2012) and increases with scale (Sistla and Schimel, 2012). Moreover, autotrophs exhibit greater variability in stoichiometric ratios than heterotrophs across the food web (Hessen et al., 2004; Persson et al., 2010; Bertrand et al., 2019).

One of the main factors influencing stoichiometric flexibility is the shift in nutrient limitation (Güsewell, 2004; Sistla and Schimel, 2012). In theory, stoichiometric homeostasis is a strategy plants use to mitigate resource limitations (Rastetter and Shaver, 1992; Wirtz and Kerimoglu, 2016). From this perspective, plants in nutrient-limited environments are expected to maintain stricter stoichiometric homeostasis (Güsewell, 2004). To achieve this, they employ various adaptive strategies. An excessively strong homeostatic mechanism could even lead to the decoupling of plants from soil. Some researchers have found that soil nutrients influence plant stoichiometric homeostasis, with plants experiencing stricter resource limitations exhibiting stronger homeostasis (Yu et al., 2011; Chen et al., 2016; Su and Shangguan, 2022). Han et al. (2011) proposed the *Stability of Limiting Elements Hypothesis*, which suggests that variability and environmental sensitivity are lowest for elements that are most limiting in nature, indicating that plant stoichiometric homeostasis varies with nutrient limitation. Chen et al. (2016) found that during the ecological restoration, as soil nutrients improved, the stoichiometry of *D. dichotoma* shifted from strong stoichiometric homeostasis in the early stage to weak stoichiometric homeostasis in the later stage. This finding also suggests that plants exhibit stronger internal homeostasis in more nutrient-restricted environments. However, this hypothesis remains unconfirmed, as the studies mentioned above did not directly address the flexibility of plants’ stoichiometric ratios are in resource-constrained environments.

The dry-hot river valley is a distinct type of river valley characterized by high temperatures, aridity, and low air humidity (Ya et al., 2004). In China, these valleys are primarily found in Yunnan, northwestern Taiwan, southwestern Hainan, and southwestern Sichuan. Within Sichuan Province, they are mainly located along the Jinsha, Yalong, and Dadu rivers in southern Ganzi, as well as the Jinsha River in Panzhihua and Liangshan (Zheng, 2010). Plant growth in these valleys is severely limited by high temperatures, arid conditions, poor soil fertility, and severe soil erosion (Shoukang et al., 2022). To optimize their fitness, plants in dry-hot river valley have evolved specific stoichiometric strategies to adapt to this environment. They typically exhibit higher leaf N and P contents and lower C:N and C:P ratios (Hong-bo et al., 2021). However, previous research has primarily focused on nutrient content and ratios, with no studies to date investigating the homeostatic stoichiometric features of plants in this region. It remains unclear whether plants enhance their adaptability through strong homeostasis. Furthermore, numerous studies have indicated that soil fertility in dry-hot valleys increases with precipitation and soil nutrient content along an elevation gradient (Lei Shan-Yu et al., 2022; Chang-ming et al., 2023) and is higher on shaded slopes compared to sunny slopes (Shoukang et al., 2022; Chang-ming et al., 2024). However, it remains uncertain whether these variations in nutrient status influence the homeostasis of plants in dry-hot valleys.

*Morus alba* is a common economic timber species in China’s dry-hot valleys, valued for both its fruit and leaves (Batiha et al., 2023). It plays a crucial role in soil and water conservation and soil improvement, exhibiting a rapid growth rate, strong drought resistance, and high environmental adaptability (Jianfeng et al., 2016; Xie et al., 2024). As a result, it has been widely planted throughout the Jinsha River valley since the 1990s (Mingqin, 1996; Sheng et al., 1999). This study examines *M. alba* by analyzing the stoichiometric characteristics of its leaves, its variation with altitude, differences between sunny and shady slopes, and correlations with soil. The objective is to determine whether plants in dry-hot valleys exhibit strong stoichiometric homeostasis and whether this homeostasis varies with altitude and slope.

## 2 Materials and methods

### 2.1 Study sites

**Figure 1:**
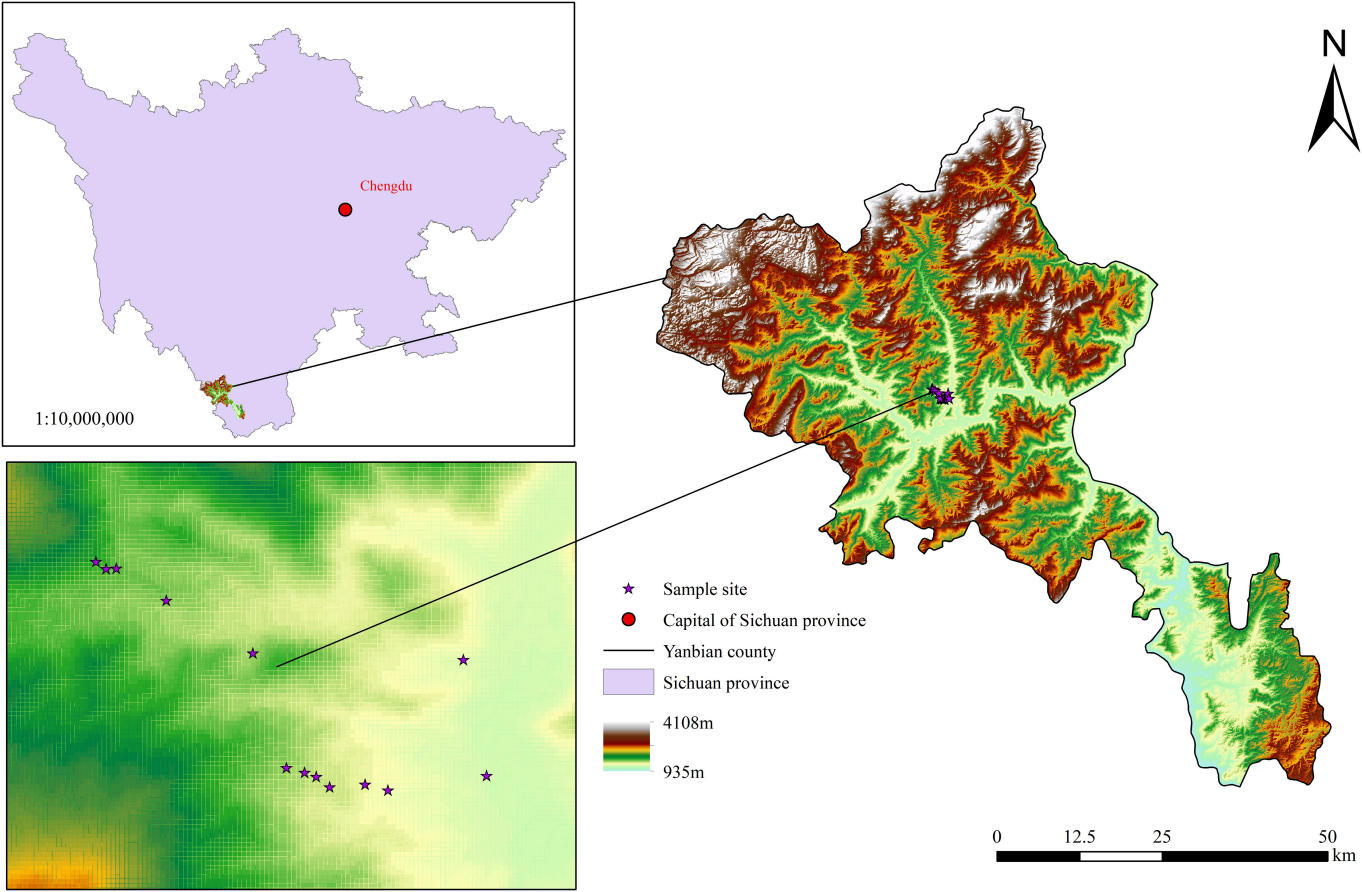
The study area.

The study area is located in Yanbian County, Panzhihua City, Sichuan Province, China (101.52°∼101.53°E, 26.95°∼26.96°N), within the dry-hot river valley at the junction of Sichuan and Yunnan provinces (**Figure 1**). This region experiences a typical South Asian tropical dry-hot valley climate, with an annual average temperature was 19.2°C. The three main soil types are bauxite, red soil, and yellow-red soil.

The predominant variety of *M. alba* cultivated in this region is Yunsang No. 2, which is used for both fruit and leaf production. In the study area, *M. alba* is primarily found at altitudes between 1,000 and 2,000 m asl, with most trees concentrated in the mulberry forest at 1,200–1,500 m asl. At altitudes of 1,000∼1,200 and 1,500∼2,000 m asl, *M. alba* was observed to be scattered along roadsides, ditches, and near houses.

### 2.2 Sampling and analysis

All samples were collected in June 2023 from eight sampling sites in mulberry orchards that had been established for more than 3 years, at altitudes ranging from 1,200 to 1,500 m asl. Sampling was conducted on both sunny and shady slopes (**Figure 1**). The mulberry trees had trunk diameters of 6–10 cm, with row spacing of 1.5–2 and plant spacing of 0.6–1 m.

At each sampling site, three 10 m^2^ × 10 m^2^ sampling plots were randomly selected. Withon each plot, three 1 m^2^ × 1 m^2^ quadrats were placed along the diagonal (at both ends and the midpoint). In each quadrat, five soil samples were collected from the center and corners at a depth of 0 to 25 cm using a soil drill, then combined into a single composite sample. Similarly, healthy *M. alba* leaf samples were collected from each quadrat.

*M. alba* leaves were oven-dried at 70°C for 48 h and then powdered. Soil samples were air-dried and subsequently ground. The ground samples were used to determine organic carbon content, total nitrogen content, and total phosphorus content using the potassium dichromate oxidation external heating method, the micro-Kjeldahl method, and the ammonium molybdate method, respectively. Measurement results are expressed as nutrient content per unit mass (g kg^−1^).

### 2.3 Statistical analyses

Statistical analyses were performed using R (version 4.4.1) and SPSS 22.0 (SPSS Inc, Chicago, USA). Variance decomposition was conducted to assess the relative effects of altitude, slope, and their interactions using the “vegan” package in R. Nutrient levels and stoichiometric ratios between sunny and shady slopes were compared using a *t*-test. To identify trends in nutrient contents and stoichiometric ratios at different altitudes, linear curve fitting was applied to examine the link between C, N, and P contents, stoichiometric ratios, and altitude. Spearman correlation analysis was used to assess the relationships between the stoichiometric ratios and C, N, and P contents.

The distribution ranges of difference values (*D*-values) for nutrient contents and stoichiometric ratios between *M. alba* leaves and soil were analyzed to determine if *M. alba* leaves maintained synchrony with soil. Firstly, the nutrient concentrations and stoichiometric ratios of mulberry leaves and soil were standardized as follows:


$$\frac{Observed\;value-Mean\;value}{Mean\;value}$$

Subsequently, the difference between the two standardized values was calculated (e.g., leaf C − soil C). If the distribution of *D*-values remained within the 95% confidence interval of its mean, it indicated that changes in leaves were synchronized with those in the soil. Conversely, if the distribution exceeded the 95% confidence interval, it was assumed that leaf changes were not synchronized with soil changes.

**Figure 2:**
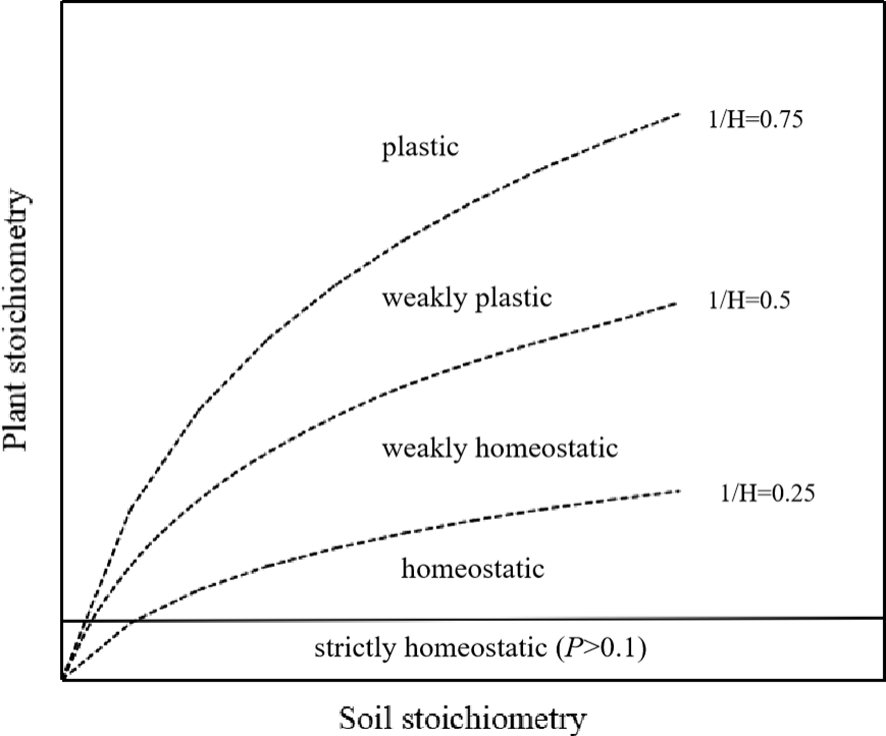
Potential patterns relating soil to plant stoichiometry (adapted from Sterner and Elser, 2002; Zhou et al., 2024).

The strength of plant stoichiometric homeostasis was analyzed on a log–log scale using the model: log(*y*) = log(*c*) + (1/*H*)log(*x*), where *y* represents the content of C, N, or P, or the ratios of C:N, C:P, or N:P in leaves; *x* represents the content of C, N, or P, or the ratios of C:N, C:P, or N:P in soil; and *c* is a constant. Values for *H* and *c* are determined through regression analysis of the relationship between *y* and *x*. The value of 1/*H* was derived from the regression slope between log *x* and log *y*, ranging from 0.00 to 1.00. To assess stoichiometric homeostasis, one-tailed tests with *α* = 0.10 were conducted. If the regression relationship was not significant (*p* > 0.10), the plant was classified as “strictly homeostatic”. However, if the regression relationship was significant (*p* < 0.10), stoichiometric homeostasis was categorized into four levels: homeostatic (0 < 1/*H* < 0.25), weakly homeostatic (0.25 < 1/*H* < 0.5), weakly plastic (0.5 < 1/*H* < 0.75), and plastic (1/*H* > 0.75) (**Figure 2**).

All figures in this paper were created using Origin 2024 (OriginLab Corporation, Northampton, MA, USA).

## 3 Results

### 3.1 Differences in the stoichiometric ratios and contents of C, N, and P in soil and *M. alba* leaves

The soil had significantly lower C, N, and P contents, as well as C:N, C:P, and N:P ratios, compared to *M. alba* leaves (*p* < 0.05). Moreover, the coefficients of variation for soil C, N, and P contents, as well as C:N, C:P, and N:P ratios, were higher than those for *M. alba* leaves (**Table 1**).

**Table 1:** Variation coefficients for stoichiometric ratios and C, N, and P contents in soil and *M. alba* leaves.

Item	Mean ± standard error	Variation coefficients
Soil C (g kg^−1^)	10.7 ± 1.31	0.49
Soil N (g kg^−1^)	1.4 ± 0.2	0.58
Soil P (g kg^−1^)	0.68 ± 0.03	0.19
Soil C:N	8.33 ± 0.39	0.19
Soil C:P	16.21 ± 2.33	0.58
Soil N:P	2.12 ± 0.37	0.7
Leaf C (g kg^−1^)	403.25 ± 3.41b	0.03
Leaf N (g kg^−1^)	29.52 ± 0.89	0.12
Leaf P (g kg^−1^)	2.79 ± 0.09	0.13
Leaf C:N	13.65 ± 0.49	0.14
Leaf C:P	146.04 ± 5.68	0.16
Leaf N:P	10.69 ± 0.36	0.13

**Figure 3:**
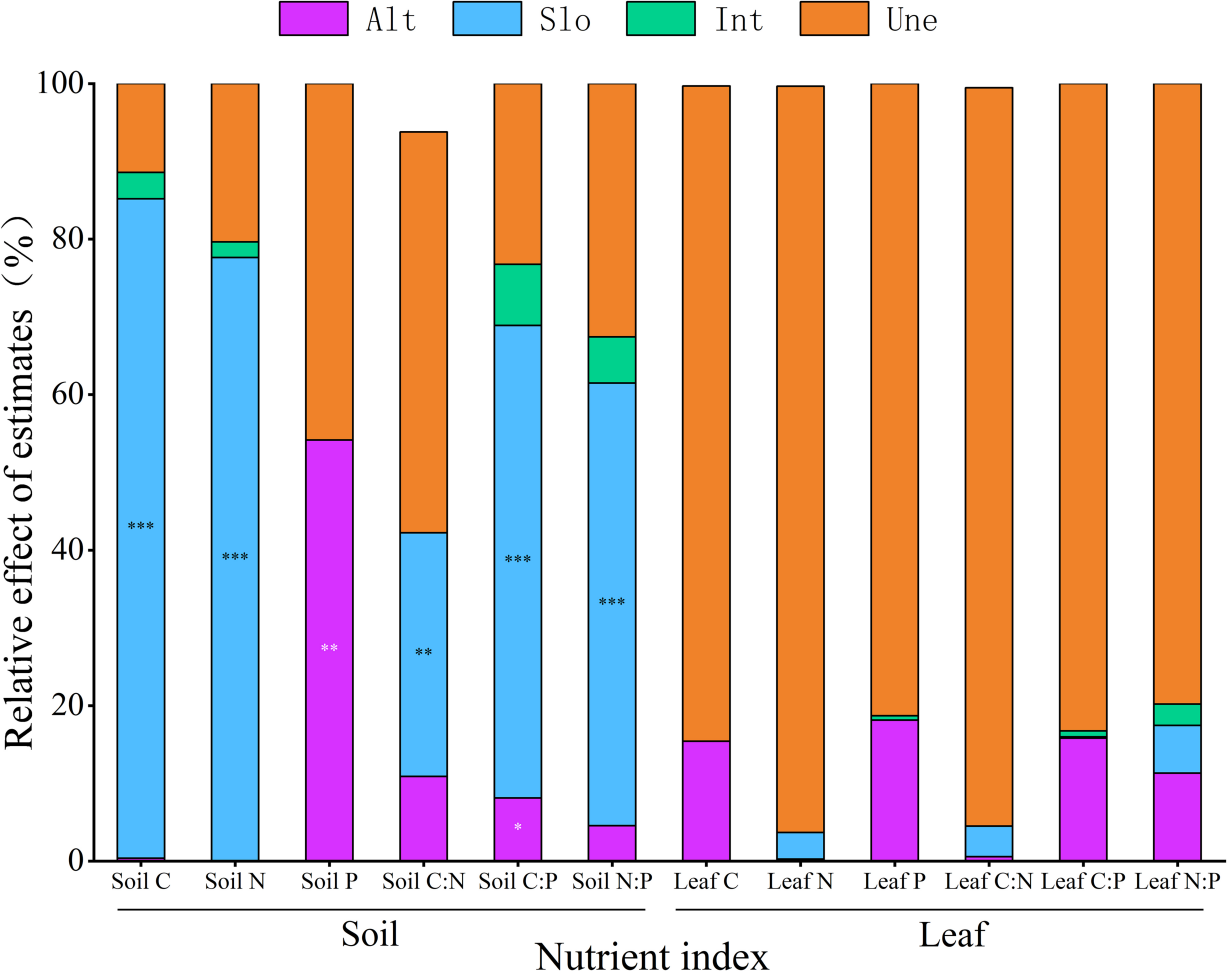
The effects of altitude and slope and their interactions on C, N, and P contents and stoichiometric ratios of soil and *M. alba* leaves. ^*^ *p* < 0.05; ^**^ *p* < 0.01; ^***^ *p* < 0.001. Alt, altitude; Slo, slope; Int, interactions; Une, unexplained.

Slope significantly influenced soil C, N, C:N, C:P, and N:P (*p* < 0.01), whereas altitude had a significant effect on soil P (*p* < 0.01). Additionally, soil C:P was influenced by both slope and altitude. Variance decomposition analysis indicated that slope was the primary factor influencing soil C, N, C:N, C:P, and N:P. For soil C, N, and N:P, the order of influence magnitude was slope > interaction effects > altitude. For soil C:N and C:P, the order of influence magnitude was slope > altitude > interaction effects. The dominant factor affecting soil P was slope (**Figure 3**).

### 3.2 C, N, and P contents and stoichiometric ratios between different slopes

**Figure 4:**
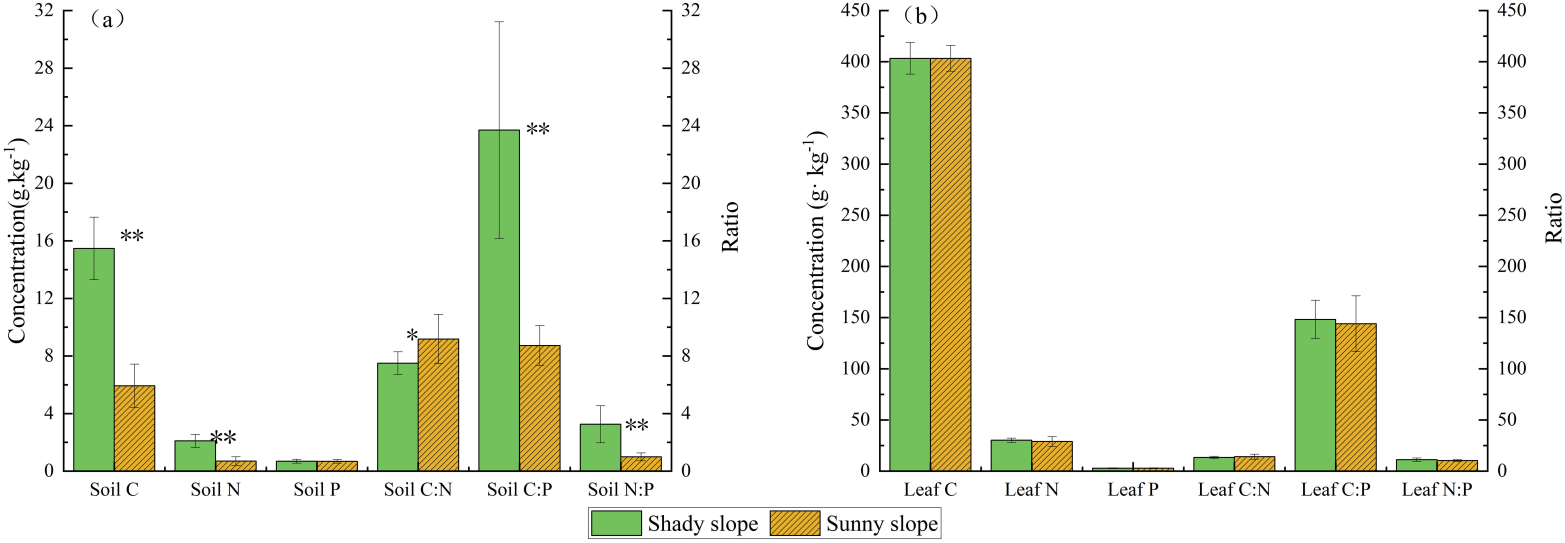
C, N, and P contents and stoichiometric ratios in soil **(a)** and *M. alba* leaves **(b)** on sunny and shady slopes. ^*^ *p* < 0.05; ^**^ *p* < 0.01;.

The nutrient content and stoichiometric ratios of the soil differed significantly between the shady and sunny slopes (*p* < 0.05). The shady slope had a higher concentration of carbon (15.47 g·kg^−1^) than the sunny slope (5.93 g·kg^−1^). Similarly, nitrogen content was significantly higher on the shady slope (2.10 g·kg^−1^) compared to the sunny slope (0.69 g·kg^−1^). In contrast, the difference in phosphorus content between the two slopes was not significant (*p* > 0.05), with the shady slope having a slightly higher concentration (0.69 g·kg^−1^) than the sunny slope (0.68 g·kg^−1^). The C:P and N:P ratios were significantly higher on the shady slope than on the sunny slope (*p* < 0.05), whereas the C:N ratio was lower (*p* < 0.05) (**Figure 4a**).

However, no significant difference was found in the stoichiometric ratios or the C, N, and P contents of *M. alba* leaves between the shady and sunny slopes (*p* > 0.05) (**Figure 4b**).

### 3.3 Responses of C, N, and P contents and stoichiometric ratios to altitude

The response of nutrient contents and stoichiometric ratios in soil and *M. alba* leaves to altitude varied significantly between sunny and shady slopes (**Table 2**).

**Table 2:** Curve fitting of nutrient contents, stoichiometric ratios, and altitude.

Item	Sunny slope				Shady slope			
	Common Slope	Slope CI (95%)	*R* ^2^	*p*-value	Common Slope	Slope CI (95%)	*R* ^2^	*p*-value
Soil C	0.1225	(0.0572, 0.1878)	0.69	< 0.05	0.0124	(− 0.1177, 0.1425)	0.01	NA
Soil N	− 0.0235	(− 0.0632, 0.0162)	0.18	NA	0.0135	(0.0002, 0.0268)	0.40	NA
Soil P	− 0.0003	(− 0.0037, 0.0031)	0.00	NA	− 0.0029	(− 0.0048, − 0.0009)	0.59	< 0.05
Soil C:N	0.0115	(− 0.0102, 0.0333)	0.15	NA	− 0.0052	(− 0.0144, 0.004)	0.17	NA
Soil C:P	0.0464	(− 0.2058, 0.2985)	0.02	NA	0.1504	(0.047, 0.2537)	0.58	< 0.05
Soil N:P	− 0.0066	(− 0.015, 0.0017)	0.29	NA	0.0154	(0.0083, 0.0225)	0.75	< 0.01
Leaf C	0.0186	(0.0079, 0.0293)	0.66	< 0.05	− 0.0146	(− 0.0224, − 0.0068)	0.69	< 0.01
Leaf N	0.0029	(− 0.0001, 0.0058)	0.38	NA	− 0.0032	(− 0.0044, − 0.002)	0.82	< 0.01
Leaf P	− 0.0009	(− 0.0018, 0)	0.39	NA	− 0.0013	(− 0.0019, − 0.0007)	0.75	< 0.01
Leaf C:N	− 0.0012	(− 0.0078, 0.0055)	0.02	NA	0.0178	(0.0106, 0.025)	0.80	< 0.05
Leaf C:P	0.0599	(0.0182, 0.1015)	0.57	< 0.05	− 0.0056	(− 0.0177, 0.0066)	0.12	NA
Leaf N:P	0.0088	(0.0004, 0.0171)	0.42	NA	− 0.0027	(− 0.0041, − 0.0013)	0.71	< 0.01

CI, confidence interval.

On the sunny slope, only soil C showed a significant linear increase with altitude (*p* < 0.05). In contrast, on the shady slope, soil P decreased linearly with altitude, while soil C:P and N:P increased (*p* < 0.05).

For *M. alba*, leaf C scaled linearly and positively with altitude on the sunny slope (slope = 0.0186, *R* ^2^ = 0.66, *p* < 0.05) but negatively on the shady slope (slope = − 0.0146, *R* ^2^ = 0.69, *p* < 0.01). Leaf N, P, and N:P decreased linearly with increasing altitude on the shady slope (*p* < 0.05) but showed no significant change on the sunny slope (*p* > 0.05). Similarly, leaf C:N increased linearly with altitude on the shady slope (*p* < 0.05) but remained relatively constant on the sunny slope (*p* > 0.05). In contrast, leaf C:P showed a positive linear relationship with altitude on the sunny slope (slope = 0.0599, *R* ^2^ = 00.57, *p* < 0.01), but no significant linear relationship was found on the shady slope (*p* > 0.05).

### 3.4 Correlations among C, N, and P contents and stoichiometric ratios

**Figure 5:**
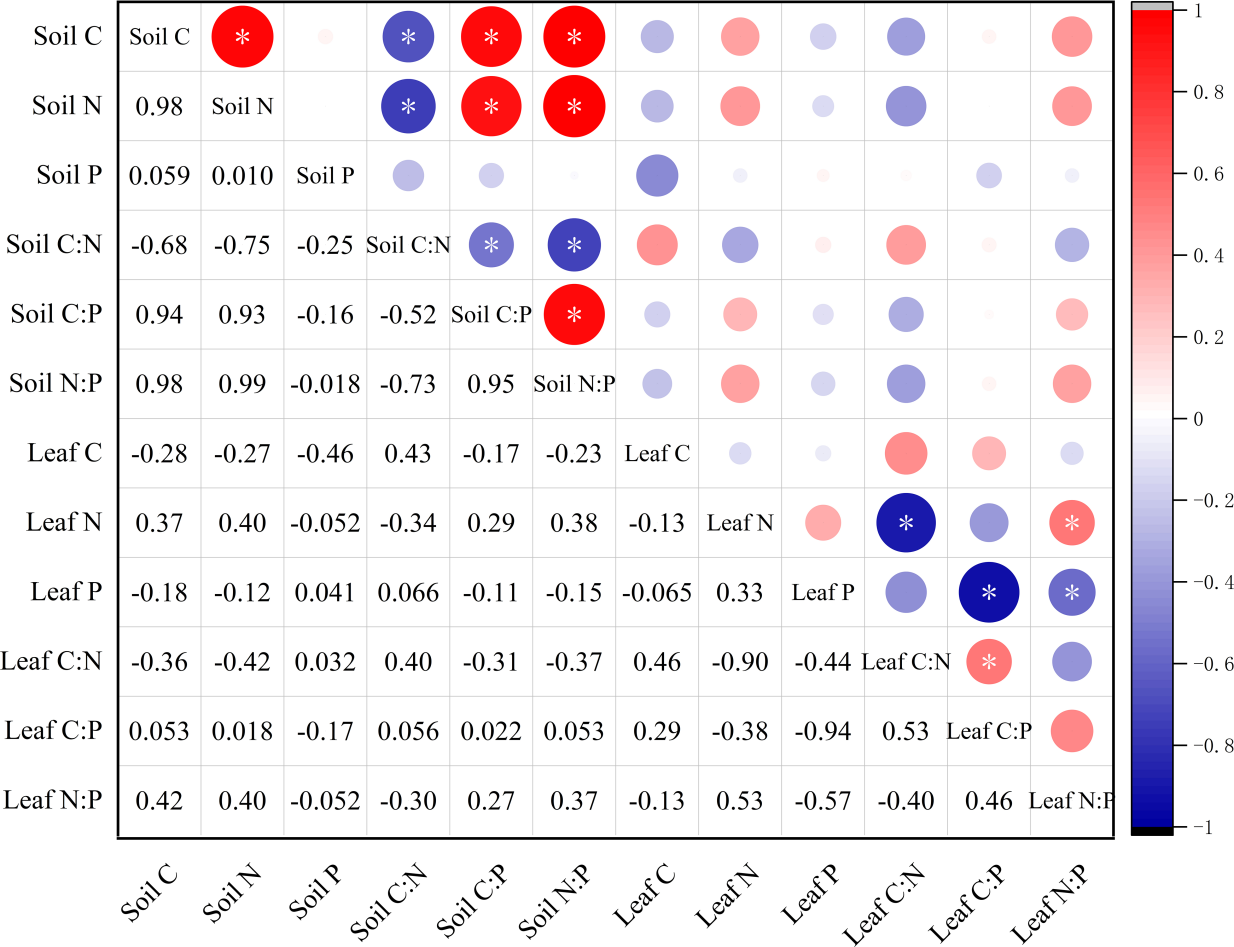
Correlations among C, N, and P contents and stoichiometric ratios in soil and *M. alba* leaves. ^*^ *p* < 0.05.

A positive correlation between was found between soil C and N (*r* = 0.98, *p* < 0.05). Additionally, both soil C and N were positively correlated with soil C:P and N:P but negatively correlated with soil C:N (*p* < 0.05). However, the correlations among leaf C, N, and P and leaf stoichiometric ratios were all found to be insignificant (*p* > 0.05). Leaf N was negatively correlated with leaf C:N, but positively correlated with leaf N:P (*p* < 0.05). Similarly, leaf P showed a negative correlation with leaf C:P but a positive correlation with N:P (*p* < 0.05). Furthermore, no significant correlation was found between soil and leaf (*p* > 0.05) (**Figure 5**).

### 3.5 *D*-values of nutrient contents and stoichiometric ratios between *M. alba* leaves and soil

**Figure 6:**
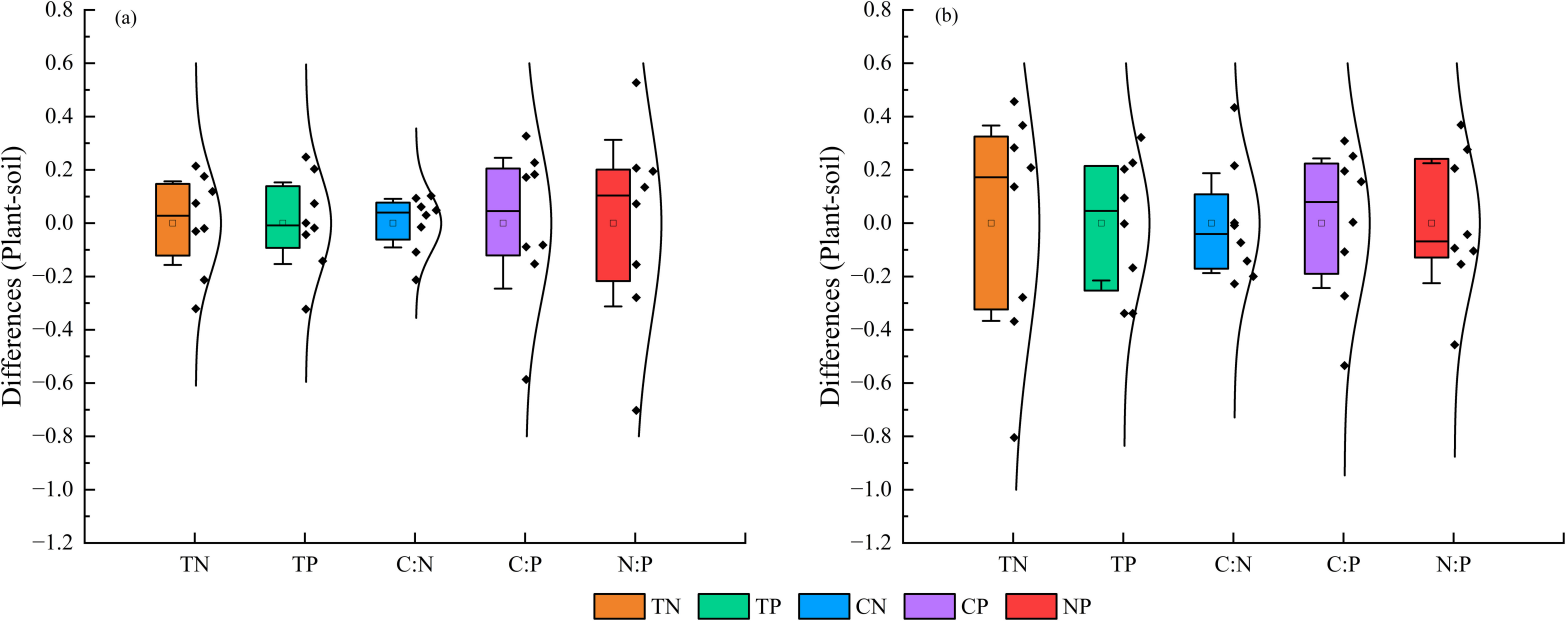
*D*-values of nutrient contents and stoichiometric ratios between *M. alba* leaves and soil on shady **(a)** and sunny **(b)** slopes. The bars above and below the box represent the 95% confidence intervals of the mean.

On both shady and sunny slopes, the *D*-values in nutrient contents and stoichiometric ratios between *M. alba* leaves and soil did not stabilize within a specific range (95% confidence interval of the means). These results suggested that the nutrient contents and stoichiometric ratios of *M. alba* leaves did not correspond to changes in soil (**Figure 6**).

### 3.6 Homeostasis on sunny and shady slopes

In this study, the regression relationships between *M. alba* leaves and soil on both shady and sunny slopes were not significant (*p* > 0.10). Therefore, *M. alba* should be classified as strictly homeostatic (**Table 3**).

**Table 3:** Homeostasis coefficients (1/*H*) between sunny and shady slopes.

Slopes	N			P			C:N			C:P			N:P		
	1/*H*	*R* ^2^	*p*-value	1/*H*	*R* ^2^	*p*-value	1/*H*	*R* ^2^	*p*-value	1/*H*	*R* ^2^	*p*-value	1/*H*	*R* ^2^	*p*-value
Shady slope	0.507	0.134	0.199	0.436	0.055	0.28	0.396	0.016	0.331	0.436	0.055	0.28	0.38	< 0.01	0.353
Sunny slope	0.166	< 0.01	0.694	− 0.317	< 0.01	0.445	0.267	< 0.01	0.522	− 0.374	< 0.01	0.361	0.235	< 0.01	0.576

*1/H*, regression slope.

## 4 Discussion

### 4.1 Nutrient limitation

The dry-hot valley has traditionally been regarded as a nutrient-poor and water-scarce environment, unsuitable for plant growth (Ya et al., 2004; Duan et al., 2017). However, in this study area, the average soil organic carbon, total soil nitrogen, and total phosphorus were 10.7, 1.4, and 0.68g·kg^−1^, respectively. These values indicate that the soil nutrient level in the study area is at a medium level compared to the national average (Fangyan et al., 2007; Tian et al., 2010; Zi-qi et al., 2022). Further analysis revealed that the soil nutrient level on the sunny slope was lower, consistent with most studies on dry-hot valleys (Guo, 2014; Guan, 2022; Hang et al., 2022; Yiting et al., 2023), whereas the nutrient level on the shady slope was at a medium level. These findings highlight the importance of distinguishing between the two slopes in future research.

Soil stoichiometry is a useful tool for understanding the cycling of elements in soil (Klemmedson and Wienhold, 1992; Zechmeister-Boltenstern et al., 2015). Soil C:N and C:P ratios reflect the rates of soil organic matter (SOM) decomposition, nutrient mineralization or immobilization, and plant nutrient limitation. A lower C:N ratio indicates a faster SOM mineralization rate, with the cumulative SOM rate being lower than the decomposition rate (Enwezor, 1976; Bui and Henderson, 2013; Pan et al., 2024). Although less frequently used, the soil C:P ratio also serves as a useful indicator of the source/nature of organic matter, with a lower C:P suggesting a higher SOM mineralization rate (Bui and Henderson, 2013). In this study, the C:N ratio in the dry-hot valley (8.33) was lower than the Chinese national average (11.9) (Tian et al., 2010; Pan et al., 2024) and findings from other studies on dry-hot valleys (Changming et al., 2022; Yiting et al., 2023; Zhifeng et al., 2024). Similarly, the C:P ratio in the study area (16.21) was significantly lower than the Chinese national average (61) (Tian et al., 2010; Pan et al., 2024) and findings from other studies on dry-hot valleys (Changming et al., 2022; Yiting et al., 2023; Zhifeng et al., 2024). These results suggest that SOM mineralization in the study area was higher than the Chinese national average and other reported dry-hot valley studies (Tian et al., 2010; Bui and Henderson, 2013).

Nitrogen and phosphorus are essential nutrients for plant growth and often serve as limiting factors in terrestrial ecosystems (Reich and Oleksyn, 2004; Yanan et al., 2014; Zhang et al., 2018). The N:P ratio in plant tissues is a widely used indicator of nutrient limitation, with values > 16 suggesting phosphorus limitation and values < 14 indicating nitrogen limitation (Güsewell, 2004; Han et al., 2005; Bertrand et al., 2019). In this study, no significant difference was observed in the N:P ratio of *M. alba* leaves between the sunny and shady slopes in the dry-hot valley, with an average of 10.28 on the sunny slope and 11.10 on the shady slope—both below 14. These findings suggest that *M. alba* growth is limited by nitrogen. The C:N and C:P ratios of plant leaves reflect nitrogen and phosphorus use efficiency as well as the plant’s capacity for carbon fixation (Sun et al., 2019; Pan et al., 2024). In this study, the C:N and C:P ratios of *M. alba* leaves in the dry-hot valley were 13.65 and 146.04, respectively, which were lower than the value reported for global (37.1, 469.2) and Chinese forest ecosystems (28.5, 513.0) (Han et al., 2005). This indicates that the plant’s nitrogen and phosphorus utilization efficiency, as well as its carbon fixation capacity, are lower in this study area compared to broader global and Chinese ecosystems (Sun et al., 2019). Additionally, reducing the C:N and C:P ratios is a known adaptive strategy that plants use to cope with resource-limited environments (Elser et al., 1996, 2000). Some species in dry-hot valleys have been observed to adopt this strategy (Lin et al., 2019; Jingwen et al., 2020; Hang et al., 2022), and *M. alba* appears to employ a similar mechanism to enhance its growth under these conditions.

### 4.2 The influences of slope and altitude on soil nutrients and stoichiometry

Slope aspect significantly influences soil nutrient levels by regulating water and energy availability and energy input, thereby shaping local abiotic and biotic environments (Bale et al., 1998; Yang et al., 2020; Qin et al., 2021). This is consistent with the findings of this study, where the primary effects on soil organic carbon, soil nitrogen, C:N, C:P, and N:P were attributed to slope differences. Previous studies have also shown that shady slopes, characterized by lower temperatures, reduced solar radiation, smaller temperature fluctuations, and higher topsoil water retention, promote organic matter accumulation, leading to more fertile compared to sunny slopes (Sharma et al., 2010; Zhaoyang et al., 2019; Tan et al., 2020; Yang et al., 2020; Liu et al., 2024). Research conducted in the dry-hot valleys of the Jinsha River (Chang-ming et al., 2024) and Minjiang River (Nan, 2016; Yang et al., 2020) similarly indicates that soil fertility is higher on shady slopes, which is consistent with the results of this study.

In mountainous areas, altitude is the primary factor driving spatial heterogeneity. Soil nutrient levels and stoichiometry vary significantly between higher and lower altitudes due to differences in climatic conditions, precipitation patterns, vegetation, and microbiome composition (Jeyakumar et al., 2020; Chang-ming et al., 2023). In this study, altitude was identified as the main factor influencing soil phosphorus content and the C:P ratio. Numerous studies have demonstrated that in dry-hot valleys, increasing altitude results in reduced dry-hot winds and higher precipitation, leading to a steady accumulation of soil organic carbon, nitrogen, and phosphorus, and ultimately enhancing soil fertility (Chunming et al., 2003; Mullen, 2011; Peng et al., 2011). However, previous studies have not thoroughly examined how soil nutrient changes with altitude differ between sunny and shady slopes. In this study, we found that soil nutrient levels responded differently to altitude depending on slope aspect. On sunny slopes, soil organic carbon exhibited a significant increasing trend with altitude, aligning with findings from other research in dry-hot valleys (Chunming et al., 2003; Mullen, 2011; Peng et al., 2011), In contrast, on shady slopes, soli phosphorus decreased with increasing altitude, indicating a reduction in soil nutrient levels. The levels of soil carbon, nitrogen, and phosphorus are influenced by various processes, including nutrient input, mineralization, immobilization, and leaching (Johnson et al., 1998; Wang et al., 2001; Soon and Arshad, 2002). As altitude increases, higher precipitation promotes vegetation growth and enhances soil organic matter accumulation (Chang-ming et al., 2024). However, unlike soil carbon and nitrogen, soil phosphorus primarily originates from the parent material rather than SOM (Tian et al., 2010). At higher altitudes, increased precipitation may lead to greater nutrient leaching, which in turn reduces soil phosphorus levels (Maojie, 2011; Liu et al., 2019). A similar decline in soil phosphorus with increasing altitude has also been observed in other studies on dry-hot valleys (Xueju, 2005; Zhen-heng and Yuan-bo, 2018). Similarly, another study found that soil nutrient levels responded differently to altitude between sunny and shady slopes, with soil nitrogen increasing with altitude on sunny slopes but decreasing on shady slopes (Zhen-heng and Yuan-bo, 2018).

### 4.3 Decoupling of nutrients and stoichiometry between *M. alba* leaves and soil

Stoichiometric homeostasis is a key parameter in ecological stoichiometry (Zhou et al., 2024). Plants with strong stoichiometric homeostasis are relatively conservative in nutrient use, whereas those with weaker homeostasis can flexibly use nutrients (Yu et al., 2010). Thus, the level of stoichiometric homeostasis reflects plant ecological adaptation mechanisms (Wirtz and Kerimoglu, 2016; Peng et al., 2017). In this study, *M. alba* exhibited strong homeostasis, as no significant correlation was found between the nutrient contents and stoichiometry ratios of *M. alba* leaves and soil. Additionally, *M. alba* leaves and soil responded differently to slope aspects and altitude. Moreover, the differences in nutrient content and stoichiometry between *M. alba* and soil were not constrained within a specific range. The 1/*H* calculation results indicated that *M. alba* was strictly homeostatic. These findings clearly support our expectation that plants in resource-limited environments, such as dry-hot valleys, exhibit strong stoichiometric homeostasis. Plants employ various metabolic and physiological mechanisms to maintain stable nutrient levels when nutrients limit their growth. The level of homeostatic flexibility largely depends on how effectively they use these limited resources (Hessen et al., 2004; Peng et al., 2017; Hong-bo et al., 2021; Su and Shangguan, 2022). Previous studies have reported that plants in dry-hot valleys can maintain stoichiometric stability by increasing nutrient absorption and resorption, which may lead to a decoupling of plant and soil stoichiometry (Jingwen et al., 2020). Plants with stronger stoichiometric homeostasis are better adapted to environmental changes (Chen et al., 2016; Peng et al., 2017; Wei et al., 2021). Our study demonstrated that *M. alba*, with its strict stoichiometric homeostasis, is well-equipped to thrive in a dry-hot environment.

## 5 Conclusions

The results of this study support the expectation that plants in dry-hot valleys exhibit strong stoichiometric homeostasis to cope with resource-limiting environments. Although the total nutrient level in the study area was at a medium level compared to the Chinese national average, the growth of *M. alba* was limited by nutrient availability, particularly nitrogen. *M. alba* maintained strict stoichiometric homeostasis on both sunny and shady slopes, despite significantly better nutrient conditions on the shady slope. This strict stoichiometric homeostasis represents an adaptive strategy of *M. alba* to the dry-hot valley, and its strength led to a decoupling of nutrient content and stoichiometry between *M. alba* leaves and the soil.

## Data Availability

The datasets presented in this article are not readily available due to restriction. Requests to access the datasets should be directed to 990221@cdnu.edu.cn.
